# Comprehensive Analysis of Sex Differences at Disease Manifestation in ANCA-Associated Glomerulonephritis

**DOI:** 10.3389/fimmu.2021.736638

**Published:** 2021-09-23

**Authors:** Désirée Tampe, Peter Korsten, Philipp Ströbel, Samy Hakroush, Björn Tampe

**Affiliations:** ^1^ Department of Nephrology and Rheumatology, University Medical Center Göttingen, Göttingen, Germany; ^2^ Institute of Pathology, University Medical Center Göttingen, Göttingen, Germany

**Keywords:** sex differences, autoimmune disease, systemic vasculitis, ANCA-associated vasculitis, ANCA glomerulonephritis

## Abstract

Anti-neutrophil cytoplasmic antibody (ANCA)-associated vasculitis (AAV) is a small vessel vasculitis affecting multiple organ systems, including the kidney. Besides investigations focusing on renal outcomes, sex differences associated with distinct clinical and histopathological findings in ANCA glomerulonephritis (GN) have not been systematically investigated. Therefore, we here aimed to systematically analyze sex differences in patients with AAV and biopsy-proven ANCA GN. We provide a comprehensive analysis of 53 kidney biopsies with ANCA GN retrospectively included between 2015 and 2020 and identified specific sex differences in ANCA GN concerning laboratory parameters and systematic scoring of renal histopathology glomerular and tubulointerstitial lesions, and extrarenal manifestations of AAV. We did not observe any correlation between sex and short-term clinical AAV course or disease severity by comparing general AAV parameters. AAV manifestations in females occurred at an older age with more joint involvement. Regarding histopathological findings, we, again, observed no sex difference among ANCA GN classification, but a significant correlation between females and distinct histopathological findings with less tubulointerstitial inflammation and vasculitis of peritubular capillaries. Finally, we here identified fewer associations between clusters of clinical, laboratory parameters, and histopathological findings in females as compared to males. These findings are of great relevance and further improve our understanding of sex differences in the pathogenesis of ANCA GN. While future studies about specific sex differences and conclusions in these clusters are crucial, our observations further support that sex differences are relevant, affect distinct parameters, and influence clinical, laboratory parameters, and histopathological findings in AAV, particularly ANCA GN.

## Introduction

According to the 2012 revised Chapel Hill Consensus Conference Nomenclature of Vasculitides, anti-neutrophil cytoplasmic antibody (ANCA)-associated vasculitis (AAV) is a small vessel vasculitis, most frequently presenting as microscopic polyangiitis (MPA) or granulomatosis with polyangiitis (GPA) (1, 2). Acute kidney injury (AKI) due to necrotizing and crescentic ANCA glomerulonephritis (GN) is a common and severe complication of AAV as it can cause progressive chronic kidney disease (CKD), end-stage kidney disease (ESKD), or death (3, 4). Several studies have investigated determinants of renal outcomes in ANCA GN, including baseline kidney function and histopathological lesions (5, 6). Proteinase 3 (PR3) and myeloperoxidase (MPO) are two major autoantigens in patients with AAV. The genes encoding these autoantigens are abnormally expressed in peripheral neutrophils of patients with active AAV (7). Mechanistically, neutrophils are activated by pathogenic ANCAs causing the release of inflammatory cytokines, reactive oxygen species and lytic enzymes, resulting in excessive formation of neutrophil extracellular traps (NETs) (8–10). Pathogenic ANCAs, in particular proteinase 3 (PR3-ANCA) and myeloperoxidase (MPO-ANCA), trigger a deleterious immune response resulting in a pauci-immune necrotizing and crescentic GN, a common manifestation of glomerular injury in AAV (11). Unlike many other autoimmune diseases, AAV has a slight predominance and higher prevalence of PR3-ANCA compared to MPO-ANCA in males (12–16). With regard to outcomes, males show a higher risk of progression to ESKD, especially in crescentic class ANCA GN (16). However, recent evidence suggests that PR3-ANCA is more prevalent than MPO-ANCA in males without any outcome differences regarding sex, potentially attributed to the known latitudinal gradient of ANCA specificity (17, 18). Besides investigations focusing on renal outcomes, sex differences in association with distinct clinical and histopathological findings in ANCA GN have not been systematically investigated (18). Therefore, we systematically analyzed sex differences in patients with biopsy-proven ANCA GN, emphasizing laboratory parameters, systematic scoring of renal histopathology including glomerular and tubulointerstitial lesions, and extrarenal manifestations of AAV.

## Methods

### Study Population

A total of 53 kidney biopsies with ANCA GN at the University Medical Center Göttingen were retrospectively included between 2015 and 2020, the patient cohort was previously described (19–25). While no formal approval was required to use routine clinical data, a favorable ethical opinion was granted by the local Ethics committee (protocol no. 22/2/14 and 28/09/17). The Birmingham Vasculitis Activity Score (BVAS) version 3 was assessed (26). Medical records were used to obtain data on age, sex, duration of disease onset before admission, diagnosis (MPA or GPA), and laboratory results including the predominant serological ANCA autoantigens (all patients were positive for MPO-ANCA or PR3-ANCA). The estimated glomerular filtration rate (eGFR) was calculated using the Chronic Kidney Disease Epidemiology Collaboration (CKD-EPI) equation (27). The simplified acute physiology score (SAPS) II was calculated according to published guidelines (28). The requirement of intensive care unit (ICU) supportive care was defined at the time of admission; all patients required critical care treatment for more than 24 hours. Renal replacement therapy (RRT) was performed intermittently in all cases. Indications for RRT included severe electrolyte and acid-base abnormalities, volume overload, or encephalopathy. Comorbidities were evaluated according to the medical records, none of the patients had type 1 diabetes mellitus or documented information about a family history of diabetes mellitus.

### Renal Histopathology

Two renal pathologists (SH and PS) independently evaluated kidney biopsies and were blinded to data analysis. Each kidney biospsy was routinely stained for periodic acid Schiff, Masson’s trichrome, silver stain, IgA, IgG and IgM to confirm pauci-immune ANCA GN, and the extent of interstitial fibrosis/tubular atrophy (IFTA) was also assessed. Furthermore, each glomerulus was scored for the presence of necrosis, crescents, and global sclerosis. Based on these scores, histopathological subgrouping according to Berden et al. into focal, crescentic, mixed, or sclerotic classes was performed (5). The ANCA renal risk score (ARRS), according to Brix et al. into low, medium, or high risk, was calculated (6). Kidney biopsies were also evaluated analogously to the Banff scoring system for allograft pathology as described previously (29). In brief, Banff score lesions included interstitial inflammation (*i*), tubulitis (*t*), arteritis (*v*), glomerulitis (*g*), interstitial fibrosis (*ci*), tubular atrophy (*ct*), arteriolar hyalinosis (*ah*), peritubular capillaritis (*ptc*), total inflammation (*ti*), inflammation in areas of IFTA (*i-IFTA*) and tubulitis in areas of IFTA (*t-IFTA*) (29). Systematic histological scoring of acute tubular injury (ATI) lesions was evaluated as previously described (30, 31). In brief, epithelial simplification and tubular dilatation, nonisometric cell vacuolization, cellular, red blood cell (RBC), and hyaline casts were given a score between 0 and 4 as a percentage of the total affected cortical area of the biopsy (score 0: <1%, 1: ≥1-10%, 2: ≥10-25%, 3: ≥25-50%, 4: >50%). In addition, infiltrates of neutrophils, eosinophils, plasma cells, and mononucleated cells (macrophages and T lymphocytes) were quantified as a fraction of the total area.

### Plasma Exchange and Remission Induction Therapy

Glucocorticoids (GCs) were administered either as intravenous pulse therapy or orally with a tapering schedule. At the time of kidney biopsy, all patients received GCs and further remission induction therapy was initiated thereafter based on histopathological confirmation of ANCA GN. Plasma exchange (PEX) was administered during the induction period at the discretion of treating physicians. Rituximab (RTX) was administered in four intravenous doses at 375 mg/m^2^ every week; RTX was not administered within 48 hours before PEX treatment. Cyclophosphamide (CYC) was administered in three intravenous doses up to 15 mg/kg every two weeks and every three weeks after that, adjusted for age and renal function. Combination therapy was administered in four intravenous doses at 375 mg/m^2^ RTX every week and two intravenous doses at 15 mg/kg CYC every two weeks. At the discretion of treating physicians, remission induction therapy depended on previous regimens and individual patient factors. RTX was preferred in younger patients, with toxicity being the main reason for this choice (32). Prophylaxis to prevent *Pneumocystis jiroveci* infection was given according to local practice.

### Statistical Methods

Variables were tested for normal distribution using the Shapiro-Wilk test. Statistical comparisons were not formally powered or prespecified. Non-normally distributed continuous variables are shown as the median and interquartile range (IQR), categorical variables are presented as frequency and percentage. For group comparisons, the Mann-Whitney U-test was used to determine differences in medians. Non-parametric between-group comparisons were performed with Pearson’s Chi-square test. Spearman’s correlation was performed to assess the correlation between clinical, laboratory, and histopathological parameters, and heatmaps reflecting the mean values of Spearman’s ρ are shown, the asterisks indicating significant correlations. Data analyses were performed with GraphPad Prism (version 8.4.3 for macOS, GraphPad Software, San Diego, California, USA). Multiple regression analyses were performed using IBM SPSS Statistics (version 27 for MacOS, IBM Corporation, Armonk, New York, USA). We retained covariates significantly associated with complement component measurements in a multivariable regression model, limiting the model covariates to avoid model over-fit. A probability (*p*) value of <*0.05* was considered statistically significant.

## Results

### Description of Demographic and Clinical Characteristics

**Figure 1:**
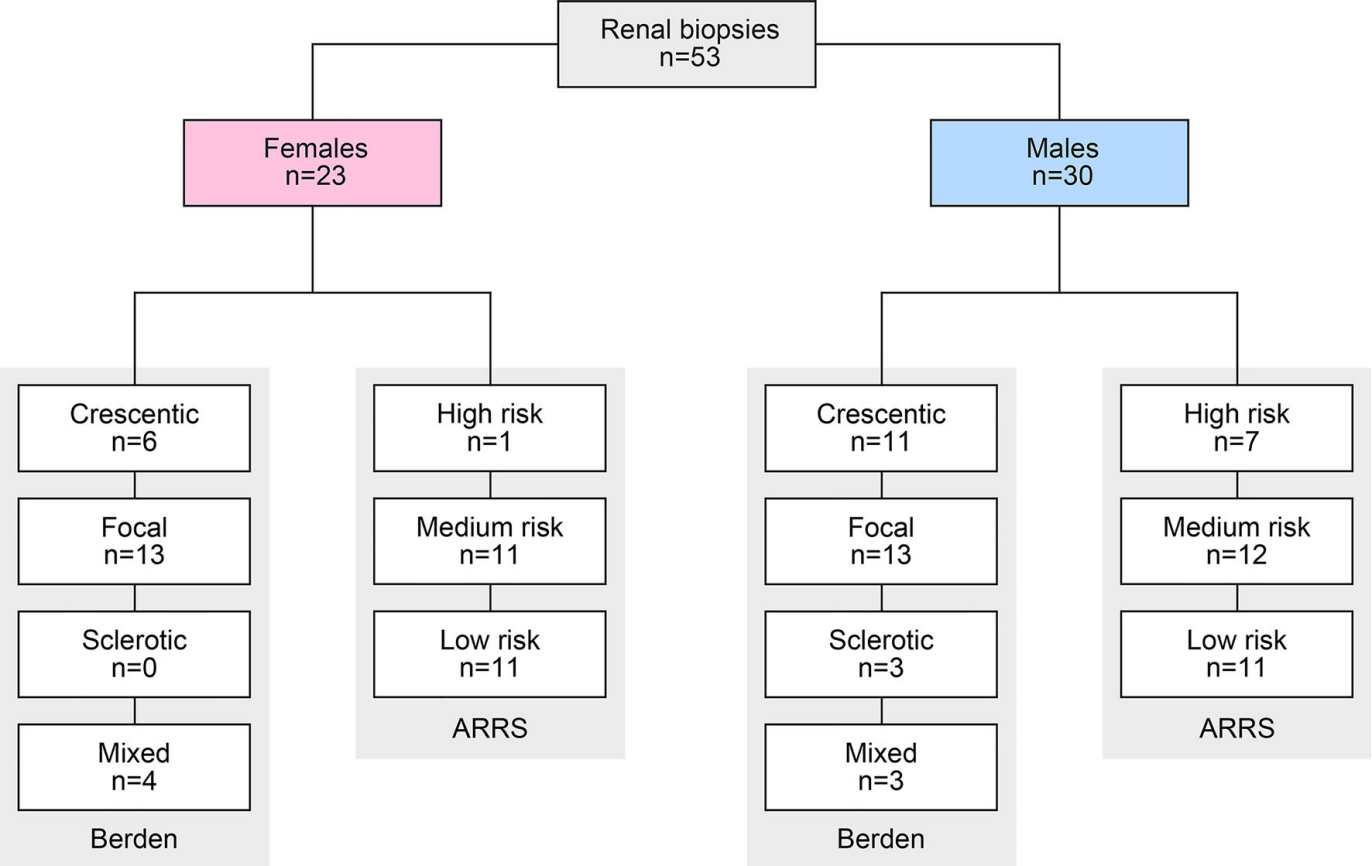
Total patient cohort of ANCA GN. STROBE flow chart of the patient disposition. ANCA, anti-neutrophil cytoplasmic antibodies; ARRS, ANCA renal risk score; GN, glomerulonephritis; STROBE, Strengthening the Reporting of Observational Studies in Epidemiology.

A total of 53 renal biopsies with ANCA GN was included. The baseline characteristics of the cohort are shown in **Table 1**. In this cohort, 23/53 (43.4%) were females, the median (IQR) age at diagnosis was 65 (54.5-74.5) years, and all patients were Caucasian. The median (IQR) disease onset before admission was 18 (7–46) days, and kidney biopsy was performed within 6 (3-9.5) days after admission to confirm renal involvement of AAV. Based on clinical characteristics, 26/53 (49.1%) patients were diagnosed as MPA, and the remainder as GPA. A total number of 8/53 (15.1%) patients had a history of vasculitis. The median (IQR) BVAS was 18 (15-20.5). The median (IQR) SAPS II at admission was 24 (19–32), and 24/53 (45.3%) of patients required ICU supportive care. There were 44/53 patients (83%) with some extrarenal manifestation of AAV (31 with lung, 9 with sinus, 12 with joint, 4 with ear, 3 with eye, 6 with peripheral nerve, and 9 with skin involvement), and 7/53 (13.2%) had alveolar hemorrhage. Based on laboratory findings, 26/53 (49.1%) positive for MPO-ANCA and 27/53 (50.1%) positive for PR3-ANCA. The worst median (IQR) eGFR at disease onset was 19 (9.7-50.2) mL/min/1.73 m^2^, and 16/53 (30.2%) required RRT within 30 days after admission. Histopathological subgrouping revealed 17/53 (43.3%) crescentic, 25/53 (49.1%) focal, 3/53 (5.7%) sclerotic, and 7/53 (13.2%) mixed class ANCA GN (5). ARRS was high in 8/53 (15.1%), intermediate in 23/53 (43.4%), and low-risk class ANCA GN in 22/53 (41.5%) of cases (**Figure 1**) (6).

**Table 1:** Clinical and laboratory parameters of the total ANCA GN cohort.

*Clinical data*	
Females – no. (%)	23 (43.4)
Median age (IQR) – years	65 (54.5-74.5)
Disease onset – median days before admission (IQR)	18 (7–46)
Kidney biopsy – median days after admission (IQR)	6 (3-9.5)
MPA/GPA subtype – no./no. (%/%)	26/27 (49.1/50.9)
History of vasculitis – no. (%)	8 (15.1)
Median SAPS II (IQR) – points	24 (19–32)
ICU supportive care – no. (%)	24 (45.3)
RRT within 30 days after admission – no. (%)	16 (30.2)
*AAV manifestations*	
Median BVAS (IQR) – points	18 (15-20.5)
Extrarenal manifestation – no. (%)	44 (83)
Lung involvement – no. (%)	31 (58.5)
Sinus involvement – no. (%)	9 (17)
Joint involvement – no. (%)	12 (22.6)
Ear involvement – no. (%)	4 (7.5)
Eye involvement – no. (%)	3 (5.7)
Nerve involvement – no. (%)	6 (11.3)
Skin involvement – no. (%)	9 (17)
Pulmonary hemorrhage – no. (%)	7 (13.2)
*Laboratory data*	
MPO-ANCA/PR3-ANCA – no./no. (%/%)	26/27 (49.1/50.9)
Median serum creatinine (IQR) – mg/dL	3.04 (1.315-4.94)
Median eGFR (IQR) – mL/min/1.73 m^2^	19 (9.7-50.2)
Median CRP (IQR) – mg/L	57.4 (19.1-106.7)
Median C3c (IQR) – g/L	1.295 (0.9925-1.413)
Median C4 (IQR) – g/L	0.26 (0.195-0.3025)
*Urinary data*	
Median uPCR (IQR) – mg/g	904.3 (505.2-1653)
Median uACR (IQR) – mg/g	445.2 (164.2-854.6)
Median α_1_-microglobulin (IQR) – mg/g	69.63 (34.8-172.5)
Median α_2_-macroglobulin (IQR) – mg/g	5.055 (2.924-11.13)
Median IgG (IQR) – mg/g	44.05 (20.5-190.8)
Hemoglobinuria – no. (%)	52 (98.1)
Acanthocytes – no. (%)	8 (15.1)
*Comorbidities*	
Arterial hypertension – no. (%)	24 (45.3)
Diabetes mellitus – no. (%)	8 (15.1)
Malignancies – no. (%)	1 (1.9)
*Histopathological subgrouping*	
Crescentic class – no. (%)	17 (43.3)
Focal class – no. (%)	26 (49.1)
Sclerotic class – no. (%)	3 (5.7)
Mixed class – no. (%)	7 (13.2)
*ARRS*	
High risk – no. (%)	8 (15.1)
Medium risk – no. (%)	23 (43.4)
Low risk – no. (%)	22 (41.5)

ANCA, anti-neutrophil cytoplasmic antibodies; BVAS, Birmingham Vasculitis Activity Score; C3c, complement factor 3 conversion product; C4, complement factor 4; CRP, C-reactive protein; eGFR, estimated glomerular filtration rate (CKD-EPI); GN, glomerulonephritis; GPA, granulomatosis with polyangiitis; ICU, intensive care unit; IQR, interquartile range; MPA, microscopic polyangiitis; MPO, myeloperoxidase; no., number; PR3, proteinase 3; RRT, renal replacement therapy; SAPS II, simplified acute physiology score II; uPCR, urinary protein-to-creatinine ratio; uACR, urinary albumin-to-creatinine ratio.

### Sex Differences Among Clinical Characteristics and Laboratory Parameters at Disease Manifestation in AAV

**Figure 2:**
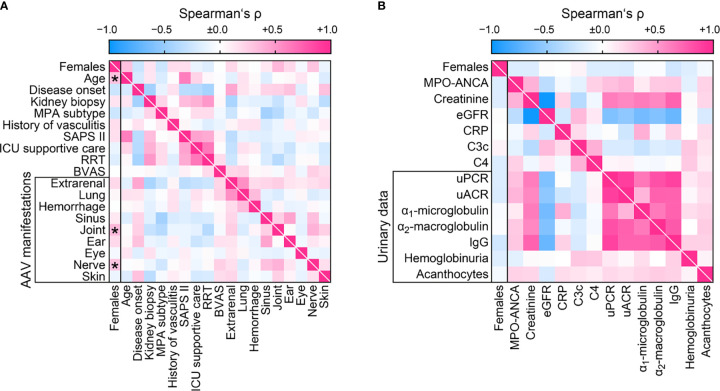
Sex differences among clinical characteristics and laboratory parameters at disease manifestation in AAV. **(A)** Sex in association with clinical findings are shown by heatmap reflecting mean values of Spearman’s ρ, asterisks indicate *p < 0.05*. **(B)** Sex in association with laboratory parameters are shown by heatmap reflecting mean values of Spearman’s ρ. AAV, ANCA-associated vasculitis; ANCA, anti-neutrophil cytoplasmic antibodies; BVAS, Birmingham Vasculitis Activity Score; C3c, complement factor 3 conversion product; C4, complement factor 4; CRP, C-reactive protein; eGFR, estimated glomerular filtration rate; IgG, immunoglobulin G; MPA, microscopic polyangiitis; MPO, myeloperoxidase; RRT, renal replacement therapy; uACR, urinary albumin-to-creatinine ratio; uPCR, urinary protein-to-creatinine ratio.

We first analyzed sex differences among clinical characteristics and laboratory parameters in AAV. We did not observe any correlation between sex and ANCA subtype, short-term clinical AAV course (disease onset, admission or time of kidney biopsy), or severity (SAPS II, need for ICU supportive care or RRT within 30 days after admission). Interestingly, females were significantly older at time of biopsy despite disease onset before admission was equally distributed (**Table 2** and **Figure 2A**), implicating that AAV manifestation in females occured at an older age. While systemic disease activity assessed by BVAS did not differ, females had significantly more joint and peripheral nerve involvement among extrarenal AAV manifestations (**Table 2** and **Figure 2A**). Multiple regression analyses confirmed that identified parameters age and joint involvement were independently attributed to females (**Table 3**). In contrast, we did not observe any sex-specific associations among systemic and urinary laboratory parameters including predominant ANCA autoantibodies (**Table 2** and **Figure 2B**). In summary, we observed no sex difference among general AAV parameters, but AAV manifestations in females occurred at an older age with more involvement of joints.

**Table 2:** Sex differences among clinical and laboratory parameters at disease onset in ANCA GN.

	Females	Males	P-value
*Clinical data*			
Median age (IQR) – years	69 (63–76)	57 (49.75-74)	***0.0291***
Disease onset – days before admission (IQR)	13 (7-24.5)	29 (13–60)	*0.0726*
Kidney biopsy – days after admission (IQR)	7 (5–9)	5 (3-10.25)	*0.3258*
MPA/GPA subtype – no./no. (%/%)	8/15 (34.8/65.2)	18/12 (60/40)	*0.0687*
History of vasculitis – no. (%)	5 (21.7)	3 (10)	*0.2367*
Median SAPS II (IQR) – points	27 (21–32)	24 (19-31.25)	*0.2693*
ICU supportive care – no. (%)	9 (39.1)	15 (50)	*0.4308*
RRT within 30 days after admission – no. (%)	5 (21.7)	11 (36.7)	*0.2407*
*AAV manifestations*			
Median BVAS (IQR) – points	18 (15–21)	18 (15-20.25)	*0.9679*
Extrarenal manifestation – no. (%)	21 (91.3)	23 (76.7)	*0.1595*
Lung involvement – no. (%)	14 (60.9)	17 (56.7)	*0.7583*
Sinus involvement – no. (%)	5 (21.7)	4 (13.3)	*0.4192*
Joint involvement – no. (%)	9 (39.1)	3 (10)	***0.0120***
Ear involvement – no. (%)	3 (13)	1 (3.3)	*0.1847*
Eye involvement – no. (%)	1 (4.4)	2 (6.7)	*0.7173*
Nerve involvement – no. (%)	5 (21.7)	1 (3.3)	***0.0361***
Skin involvement – no. (%)	3 (13)	6 (20)	*0.5038*
Pulmonary hemorrhage – no. (%)	3 (13)	4 (13.3)	*0.9754*
*Laboratory data*			
MPO-ANCA/PR3-ANCA – no./no. (%/%)	9/14 (39.1/60.9)	17/13 (56.7/43.3)	*0.2056*
Median serum creatinine (IQR) – mg/dL	1.97 (1.29-3.94)	3.64 (1.318-5.44)	*0.2261*
Median eGFR (IQR) – mL/min/1.73 m^2^	25.3 (12.5-44.2)	16.35 (9.35-54.3)	*0.9539*
Median CRP (IQR) – mg/L	47.1 (20.5-109.3)	63.7 (16-108.1)	*0.9752*
Median C3c (IQR) – g/L	1.3 (1.093-1.418)	1.27 (0.895-1.413)	*0.7313*
Median C4 (IQR) – g/L	0.245 (0.163-0.293)	0.29 (0.195-0.35)	*0.2801*
*Urinary data*			
Median uPCR (IQR) – mg/g	819.6 (556.9-1492)	1339 (495.3-3020)	*0.2143*
Median uACR (IQR) – mg/g	445.2 (99.-697.2)	486.3 (211.2-1763)	*0.1888*
Median α_1_-microglobulin (IQR) – mg/g	69.63 (28.4-150.3)	78.45 (34.95-185.8)	*0.8137*
Median α_2_-macroglobulin (IQR) – mg/g	4.935 (2.95-10.12)	5.139 (2.84-13.01)	*0.8719*
Median IgG (IQR) – mg/g	32.98 (19.2-81.2)	102.2 (20.1-248.4)	*0.0976*
Hemoglobinuria – no. (%)	22 (95.7)	30 (100)	*0.2489*
Acanthocytes – no. (%)	3 (13)	5 (16.7)	*0.7150*
*Comorbidities*			
Arterial hypertension – no. (%)	11 (47.8)	13 (43.3)	*0.7447*
Diabetes mellitus – no. (%)	3 (13)	5 (16.7)	*0.7150*
Malignancies – no. (%)	1 (4.3)	0 (0)	*0.2489*

For group comparisons, the Mann-Whitney U-test was used to determine differences in medians. In addition, non-parametric between-group comparisons were performed with Pearson’s Chi-square test. Bold indicates statistically significant values at group level.

ANCA, anti-neutrophil cytoplasmic antibodies; BVAS, Birmingham Vasculitis Activity Score; C3c, complement factor 3 conversion product; C4, complement factor 4; CRP, C-reactive protein; eGFR, estimated glomerular filtration rate (CKD-EPI); GN, glomerulonephritis; GPA, granulomatosis with polyangiitis; ICU, intensive care unit; IQR, interquartile range; MPA, microscopic polyangiitis; MPO, myeloperoxidase; no., number; PR3, proteinase 3; RRT, renal replacement therapy; SAPS II, simplified acute physiology score II; uPCR, urinary protein-to-creatinine ratio; uACR, urinary albumin-to-creatinine ratio.

**Table 3:** Multiple regression analyses of parameters attributed to females.

	ß	SE	P-value
*Parameter*			
Age – years	0.2849	0.0043	***0.0276***
Joint involvement	0.2751	0.1603	***0.0475***
Nerve involvement	0.1778	0.2117	*0.1951*

Bold indicates statistically significant values.

SE, standard error.

### Sex Differences Among Histopathological Findings at Disease Onset and Choice of Remission Induction Therapy in ANCA GN

**Figure 3:**
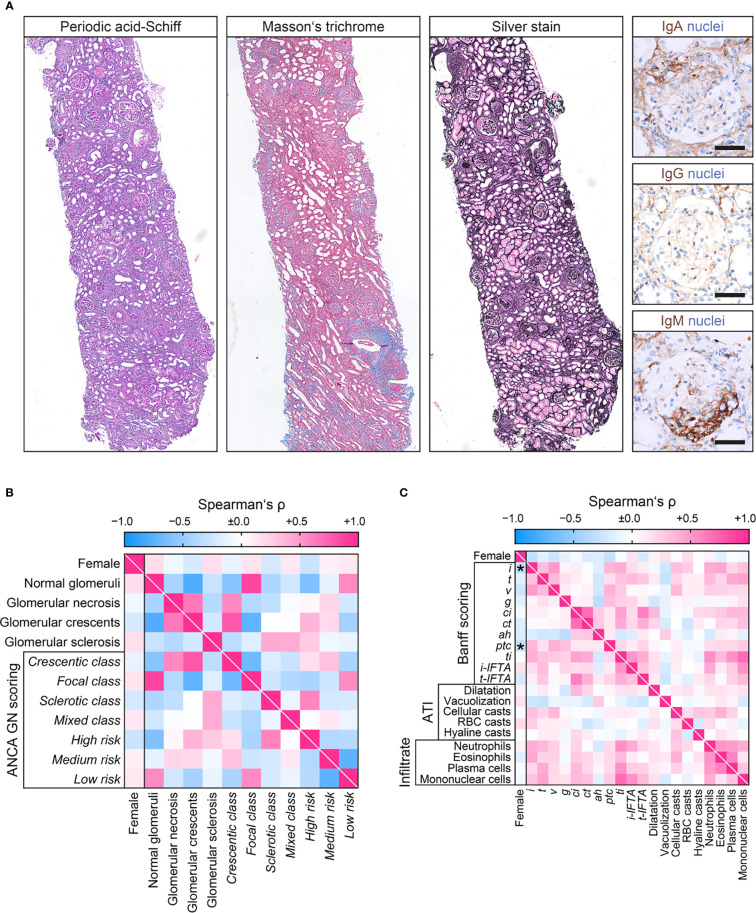
Sex differences among histopathological findings at disease manifestation in ANCA GN. **(A)** Representative photomicrographs of pauci-immune ANCA GN showing crescent formations with IgM entrapment within cellular proliferations and injured glomerular capilaries. Note, lack of granular or linear staining of IGA and IgA along the GBM typical for pauci-immune GN (scale bars: 50 µm). **(B)** Sex in association with glomerular lesions and ANCA GN scoring are shown by heatmap reflecting mean values of Spearman’s ρ. **(C)** Sex in association with tubulointerstitial lesions analogous to the Banff scoring system, ATI lesions, and distinct inflammatory infiltrates are shown by heatmap reflecting mean values of Spearman’s ρ, asterisks indicate p<0.05. ah, arteriolar hyalinosis; ANCA, anti-neutrophil cytoplasmic antibodies; ATI, acute tubular injury; ci, interstitial fibrosis; ct, tubular atrophy; g, glomerulitis; GN, glomerulonephritis; i, interstitial inflammation; i-IFTA, inflammation in IFTA; RBC, red blood cell; t, tubulitis; ptc, peritubular capillaritis; ti, total inflammation; t-IFTA, tubulitis in IFTA; v, intimal arteritis.

We next analyzed sex differences among histopathological findings in pauci-immune ANCA GN (**Figure 3A**). The number of normal glomeruli, glomerular necrosis, crescents, or sclerosis did not differ with regard to sex, also reflected by ANCA GN scoring (**Table 4** and **Figure 3B**) (5, 6). Interestingly, females had significantly less interstitial inflammation (*i*) and peritubular capillaritis (*ptc*) among tubulointerstitial lesions according to the Banff scoring system (**Figure 3C**) (29). In contrast, we did not observe an association between sex and ATI lesions or inflammatory infiltrates (**Table 4** and **Figure 3C**) (30, 31). Furthermore, choice of PEX and remission induction therapy did not differ with regard to sex (**Table 5**). In summary, we observed no sex differences among general ANCA GN scores or choice of remission induction therapy. Interestingly, there was a significant correlation with distinct histopathological findings including fewer interstitial inflammation and vasculitis manifestation in peritubular capillaries in females.

**Table 4:** Sex differences among histopathological findings at disease onset in ANCA GN.

	Females	Males	P-value
*Glomerular lesions*			
Median total glomeruli (IQR) – no.	19 (11–33)	14.5 (10.75-27)	*0.1721*
Median normal glomeruli (IQR) – %	54.55 (35.29-75.76)	43.43 (11.6-72.88)	*0.2365*
Median glomerular necrosis (IQR) – %	12.5 (0-42.86)	16.67 (0-51.39)	*0.5919*
Median glomerular crescents (IQR) – %	27.27 (8.33-43.75)	41.8 (11.88-63.4)	*0.1581*
Median glomerular sclerosis (IQR) – %	12.5 (0–30)	1.85 (0-19.41)	*0.2704*
*Histopathological subgrouping*			
Crescentic class – no. (%)	6 (26.1)	11 (36.7)	*0.2892*
Focal class – no. (%)	13 (56.5)	13 (43.3)	
Sclerotic class – no. (%)	0 (0)	3 (10)	
Mixed class – no. (%)	4 (17.4)	3 (10)	
*ARRS*			
High risk – no. (%)	1 (4.3)	7 (23.3)	*0.1586*
Medium risk – no. (%)	11 (47.8)	12 (40)	
Low risk – no. (%)	11 (47.8)	11 (36.7)	

For group comparisons, the Mann-Whitney U-test was used to determine differences in medians. In addition, non-parametric between-group comparisons were performed with Pearson’s Chi-square test.

ANCA, anti-neutrophil cytoplasmic antibodies; ARRS, ANCA renal risk score; GN, glomerulonephritis; IQR, interquartile range; no., number.

**Table 5:** Sex differences in choice of PEX and remission induction therapy in ANCA GN.

	Females	Males	P-value
*PEX therapy*			
PEX – no. (%)	6 (26.1)	14 (46.7)	*0.1255*
Median sessions of PEX (IQR) – no.	5 (4.75-7.25)	5 (5–5)	*0.5236*
*Remission induction therapy*			
RTX – no. (%)	7 (30.4)	11 (36.7)	*0.3668*
CYC – no. (%)	13 (56.5)	12 (40)	
RTX/CYC – no. (%)	2 (8.7)	6 (20)	

For group comparisons, the Mann-Whitney U-test was used to determine differences in medians. In addition, non-parametric between-group comparisons were performed with Pearson’s Chi-square test.

ANCA, anti-neutrophil cytoplasmic antibodies; CYC, cyclophosphamide; GCs, glucocorticoids; IQR, interquartile range; no., number; PEX, plasma exchange; RTX, rituximab.

### Sex-Specific Cluster Analysis for the Association Between Clinical, Laboratory Parameters and Histopathological Findings at Disease Manifestation in ANCA GN

**Figure 4:**
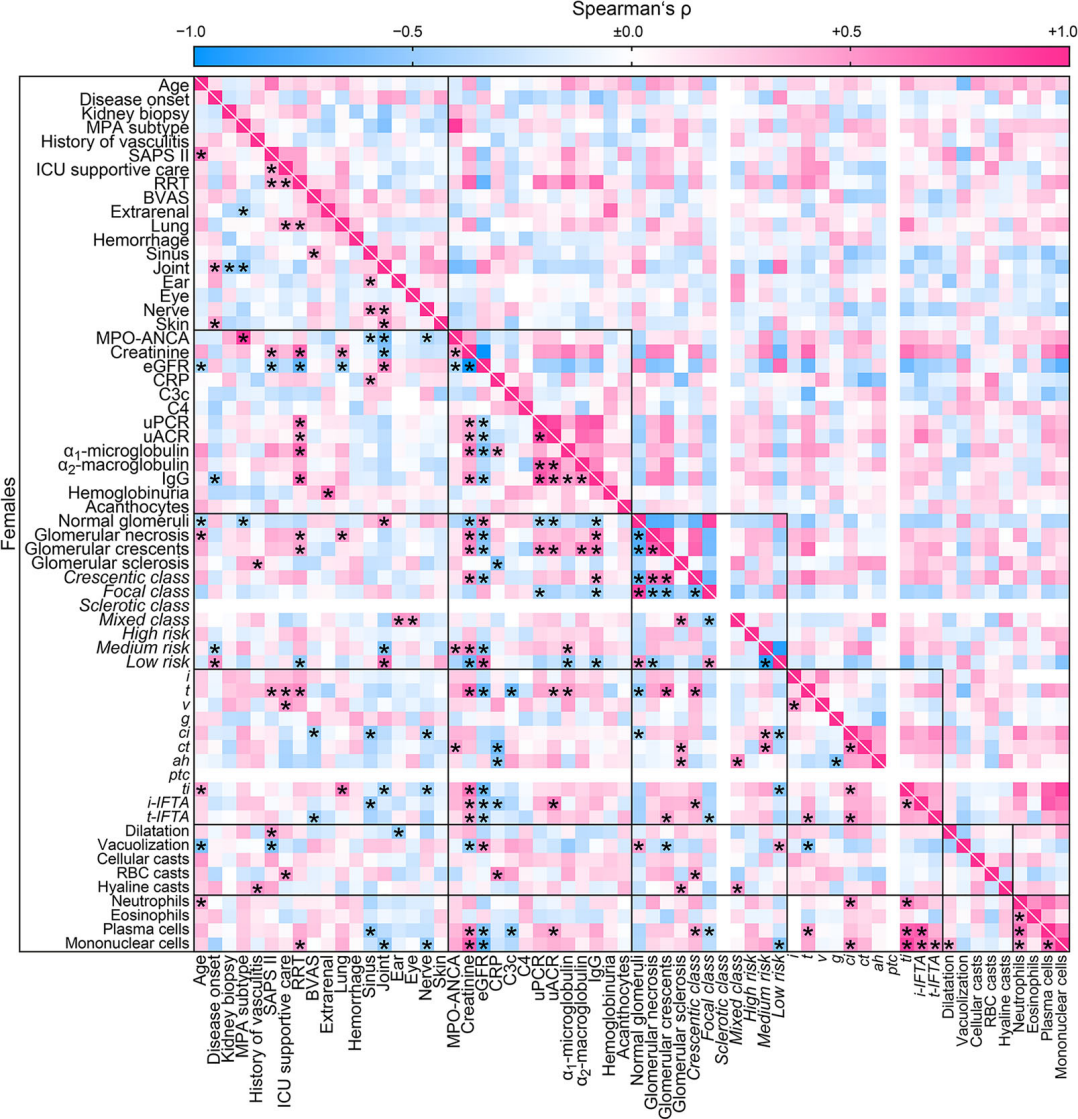
Female-specific cluster analysis for the association between clinical, laboratory parameters, and histopathological findings in ANCA GN. Associations of clinical, laboratory parameters, and histopathological clusters in ANCA GN specifically in females are shown by heatmap reflecting mean values of Spearman’s ρ, asterisks indicate *p < 0.05*. Clusters are depicted by black boxes, the empty lines for sclerotic class ANCA GN and *ptc* within the heatmap reflect no data analysis because the respective parameters were absent in all cases. ah, arteriolar hyalinosis; ANCA, anti-neutrophil cytoplasmic antibodies; ATI, acute tubular injury; BVAS, Birmingham Vasculitis Activity Score; C3c, complement factor 3 conversion product; C4, complement factor 4; ci, interstitial fibrosis; CRP, C-reactive protein; ct, tubular atrophy; eGFR, estimated glomerular filtration rate; g, glomerulitis; GN, glomerulonephritis; i, interstitial inflammation; IgG, immunoglobulin G; i-IFTA, inflammation in IFTA; MPA, microscopic polyangiitis; MPO, myeloperoxidase; RBC, red blood cell; RRT, renal replacement therapy; t, tubulitis; ptc, peritubular capillaritis; ti, total inflammation; t-IFTA, tubulitis in IFTA; uACR, urinary albumin-to-creatinine ratio; uPCR, urinary protein-to-creatinine ratio; v, intimal arteritis.

**Figure 5:**
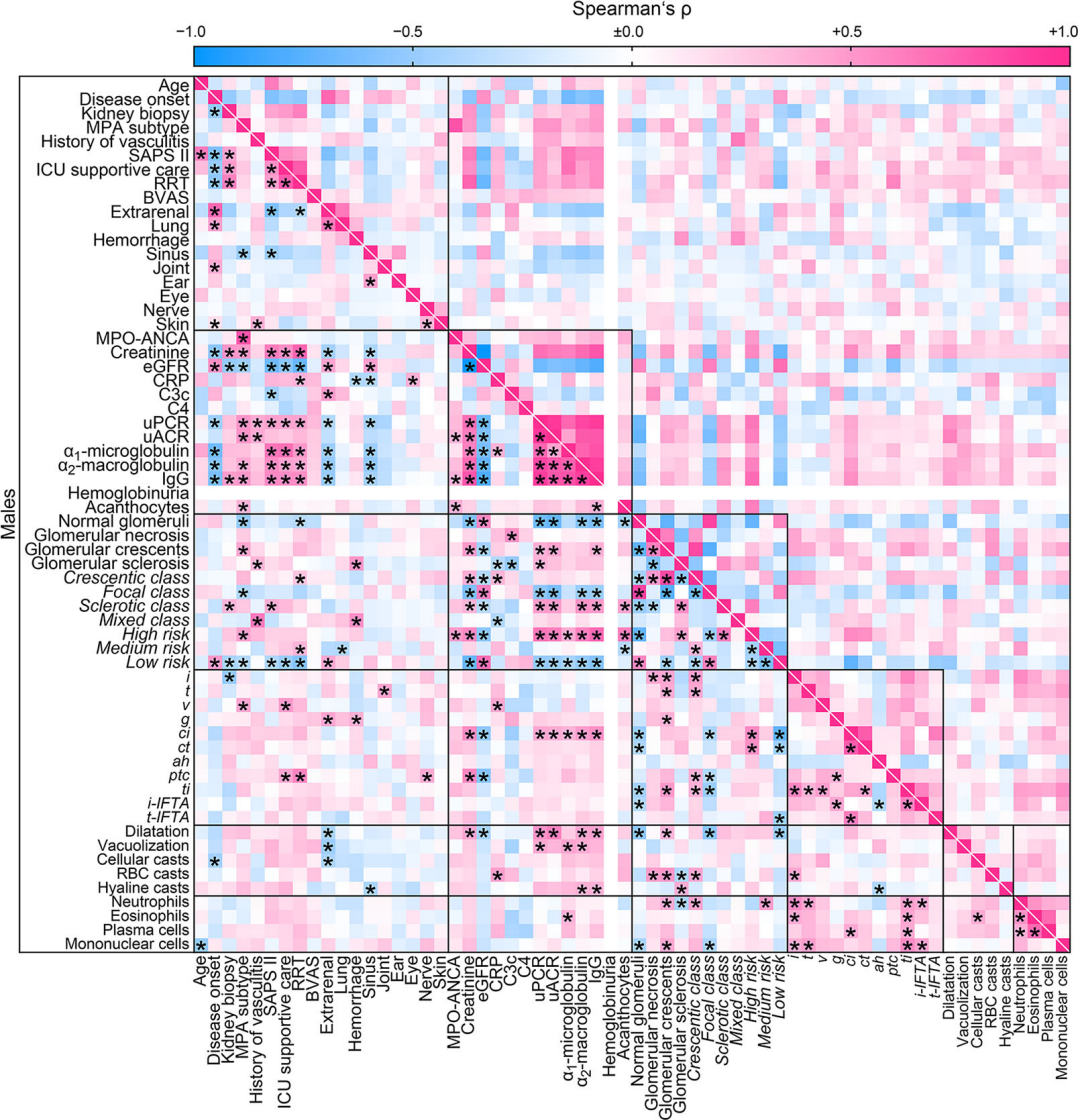
Male-specific cluster analysis for the association between clinical, laboratory parameters, and histopathological findings in ANCA GN. Associations of clinical, laboratory parameters, and histopathological clusters in ANCA GN specifically in males are shown by heatmap reflecting mean values of Spearman’s ρ, asterisks indicate *p < 0.05*. Clusters are depicted by black boxes, the empty line for hemoglobinuria within the heatmap reflects no data analysis because the respective parameter was present in all cases. *ah*, arteriolar hyalinosis; ANCA, anti-neutrophil cytoplasmic antibodies; ATI, acute tubular injury; BVAS, Birmingham Vasculitis Activity Score; C3c, complement factor 3 conversion product; C4, complement factor 4; *ci*, interstitial fibrosis; CRP, C-reactive protein; *ct*, tubular atrophy; eGFR, estimated glomerular filtration rate; *g*, glomerulitis; GN, glomerulonephritis; *i*, interstitial inflammation; IgG, immunoglobulin G; *i-IFTA*, inflammation in IFTA; MPA, microscopic polyangiitis; MPO, myeloperoxidase; RBC, red blood cell; *t*, tubulitis; *ptc*, peritubular capillaritis; RRT, renal replacement therapy; *ti*, total inflammation; *t-IFTA*, tubulitis in IFTA; uACR, urinary albumin-to-creatinine ratio; uPCR, urinary protein-to-creatinine ratio; *v*, intimal arteritis.

Finally, we aimed to identify sex-specific associations between clinical, laboratory, and histopathological parameters in ANCA GN by a separate analysis of females and males. Overall, we identified a significant association between 208/3844 (5.4%) parameters included in females (**Figure 4**) compared to 302/3844 (7.9%) parameters in males (**Figure 5**). The decreased association in females was attributed to less correlation between clusters of clinical, laboratory parameters, glomerular lesions, and ANCA GN scoring *versus* all other included parameters (**Table 6**). Notably, there was a less robust association of the cluster between serological and clinical parameters and scoring of glomerular lesions in ANCA GN (**Figures 4**, **5**). In addition, there was a low correlation between the cluster of glomerular scoring and tubulointerstitial lesions in ANCA GN (**Figures 4**, **5**). Thus, we identified a lesser, female-specific association between clusters of clinical and serological parameters and histopathological findings in ANCA GN.

**Table 6:** Sex differences among clusters of clinical, laboratory parameters, and histopathological findings in ANCA GN.

	Females	Males	P-value
Clinical parameters *versus* other – no. (%)	59 (7.4)	90 (11.4)	***0.0076***
Laboratory parameters *versus* other – no. (%)	73 (11.3)	127 (19.6)	***<0.0001***
Glomerular lesions and ANCA GN scoring *versus* other – no. (%)	66 (11.8)	108 (19.3)	***0.0005***
Tubulointerstitial lesions *versus* other – no. (%)	53 (7)	54 (7.1)	*0.9201*
ATI lesions *versus* other – no. (%)	17 (5.8)	29 (9.8)	*0.0654*
Inflammatory infiltrates *versus* other – no. (%)	24 (10.3)	22 (9.5)	*0.7560*

Non-parametric between-group comparisons were performed with Pearson’s Chi-square test. Bold indicates statistically significant values at group level.

ANCA, anti-neutrophil cytoplasmic antibodies; ATI, acute tubular injury; GN, glomerulonephritis; no., number.

## Discussion

We here provide a comprehensive analysis and identified specific sex differences in ANCA GN concerning serologic parameters, systematic scoring of renal histopathology including glomerular and tubulointerstitial lesions, and extrarenal manifestations of AAV. Comparing general AAV parameters, we did not observe any correlation between sex and short-term clinical AAV course or severity. In our cohort, AAV manifestations in females occurred at an older age, as reported previously (33, 34). In addition, we observed more involvement of joints and peripheral nerves in females. Regarding histopathological findings, we, again, observed no sex differences among general ANCA GN scoring but a significant correlation with distinct histopathological findings including less tubulointerstitial inflammation in females.

Based on previous observations, male patients with ANCA GN had a significantly higher risk of progression to ESKD than females in a Norwegian cohort of patients with ANCA GN (16). The most crucial sex difference has been reported in crescentic class ANCA GN, representing active glomerular lesions and supporting the concept that observed outcome differences are caused by sex-specific inflammatory differences and responses to immunosuppressive therapy. In contrast, no significant sex-specific difference in ANCA GN outcomes has been observed when combining ESKD and death as a composite outcome in Irish and British patients with ANCA GN (18). These observations might potentially be attributed to the known latitudinal gradient of ANCA specificity (17, 18). Our observation of sex-specific differences in tubulointerstitial inflammation is relevant since tubulointerstitial inflammation has previously been associated with active glomerular lesions (35). In addition, interstitial inflammation is more pronounced in MPO-ANCA than in PR3-ANCA GN, further supporting the hypothesis that interstitial lesions differ between ANCA GN subtypes (20, 35). In the current study, we did not observe sex-specific differences with regard to the ANCA subtype. Still, less interstitial inflammation in females was observed, further supporting that sex may affect AAV manifestations and outcomes. While only limited data are available, distinct inflammatory lesions have previously been shown to affect the long-term renal outcomes in ANCA GN (36).

In addition, we observed fewer vasculitis manifestations in peritubular capillaries in females with ANCA GN. The prevalence of interstitial vasculitis manifestations has been described in a considerable subset of patients with ANCA GN ranging from 10 to 35% (35, 37–41). Generally, histopathological subgrouping of ANCA GN into four classes (focal, crescentic, mixed, and sclerotic) as defined by Berden et al. in 2010 was proposed to predict long-term renal survival rates (5). However, unlike Berden’s classification, Brix et al. in 2018 suggested the ANCA renal risk score (ARRS) by incorporation of baseline glomerular filtration rate (GFR) to the histopathological findings (percentage of normal glomeruli, tubular atrophy/interstitial fibrosis) to predict ESKD in patients with AAV (6). Recently, interstitial vasculitis has been shown to improve long-term outcome prediction in ANCA GN in both scoring systems (42). These observations underscore the pathogenic role of interstitial vasculitis in ANCA GN, and our findings of more minor vasculitis manifestations in peritubular capillaries in females further improve our understanding of sex differences in AAV.

Finally, we identified a less pronounced association between clusters of clinical and laboratory parameters and histopathological findings in ANCA GN in females. There was a less robust association of the cluster of serological with clinical parameters and scoring of glomerular lesions in ANCA GN in our cohort. In addition, there was a low correlation between the cluster of glomerular scoring and tubulointerstitial lesions in ANCA GN. While future studies regarding specific sex differences in these clusters are crucial, these observations further support that sex differences affect distinct parameters. Furthermore, they suggest an interplay between clinical, laboratory parameters, and histopathological findings in AAV, particularly in ANCA GN.

The main limitations of our study are its retrospective design, the small patient number, and no long-term follow-up data on renal outcomes. Furthermore, we here aimed to sex-specific data by clustering associative data with regard of clinical, laboratory parameters, and histopathological findings in ANCA GN, requiring further investigation with regard to specific parameters. Nevertheless, we here provide a comprehensive analysis and identified specific sex differences in ANCA GN regarding laboratory parameters, systematic scoring of renal histopathology including glomerular and tubulointerstitial lesions, and extrarenal manifestations of AAV.

## Data Availability Statement

The original contributions presented in the study are included in the article/supplementary material. Further inquiries can be directed to the corresponding authors.

## Ethics Statement

The studies involving human participants were reviewed and approved by the Institutional Review Board of the University Medical Center Göttingen, Germany. The patients/participants provided their written informed consent to participate in this study.

## Author Contributions

BT conceived the study, collected and analyzed data, and wrote the first draft. SH and DT collected and analyzed data. SH and PS evaluated histopathological findings. PK analyzed data and edited the manuscript. All authors contributed to the article and approved the submitted version.

## Funding

This research was funded by the Research program, University Medical Center, University of Göttingen, grant number 1403720. This research was also funded by the German Research Foundation, KFO (CRU) 5002, grant number STR 638/3-1 (DFG). We also acknowledge support from the Open Access Publication Funds of the Göttingen University. The funders had no role in the study’s design, in the collection, analyses, or interpretation of data, in the writing of the manuscript, or in the decision to publish the results.

## Conflict of Interest

The authors declare that the research was conducted in the absence of any commercial or financial relationships that could be construed as a potential conflict of interest.

## Publisher’s Note

All claims expressed in this article are solely those of the authors and do not necessarily represent those of their affiliated organizations, or those of the publisher, the editors and the reviewers. Any product that may be evaluated in this article, or claim that may be made by its manufacturer, is not guaranteed or endorsed by the publisher.
